# Differential Transmission of Antiviral Drug-Resistant Chikungunya Viruses by *Aedes* Mosquitoes

**DOI:** 10.1128/mSphere.00230-18

**Published:** 2018-08-22

**Authors:** Leen Delang, Pei-Shi Yen, Thomas Vallet, Marie Vazeille, Marco Vignuzzi, Anna-Bella Failloux

**Affiliations:** a Department of Microbiology and Immunology, Rega Institute for Medical Research, Laboratory of Virology and Chemotherapy, KU Leuven-University of Leuven, Leuven, Belgium; b Laboratory of Arboviruses and Insect Vectors, Institut Pasteur, Paris, France; c Viral Populations and Pathogenesis Unit, Centre National de la Recherche Scientifique UMR 3569, Institut Pasteur, Paris, France; CDC

**Keywords:** *Aedes*, antiviral agents, chikungunya virus, drug resistance, mosquito, transmission

## Abstract

The chikungunya virus (CHIKV) is transmitted by female Aedes aegypti and Aedes albopictus mosquitoes, mostly present in (sub)tropical regions. No antivirals are available to treat CHIKV infections. If antiviral drugs are proven efficient to treat CHIKV-infected patients, it will be pivotal to determine whether drug-resistant viruses can be transmitted from one human to another by their mosquito vectors. We orally infected Aedes aegypti mosquitoes with a blood meal containing wild-type or drug-resistant CHIKV variants (i.e., MADTP^res^ CHIKV, with mutation in the nsP1 gene, and T-705^res^ CHIKV, with mutation in the RNA-dependent RNA polymerase [RdRp] gene). Viral loads were quantified in bodies (infection), heads (dissemination), and saliva (transmission) of individual mosquitoes. The infection rate of the resistant viruses was similar to that of the wild-type virus. However, the dissemination of T-705^res^ CHIKV was markedly decreased compared to wild-type and MADTP^res^ CHIKV. Furthermore, T-705^res^ CHIKV was only transmitted in the saliva at day 20 postinfection (p.i.), whereas transmission of wild-type CHIKV was observed at day 3 p.i. The attenuated phenotype of the T-705^res^ virus was confirmed in mosquito cell culture, whereas the replication fitness in Vero cells was similar to that of the wild type. In bodies and heads of mosquitoes infected with the resistant variants, the resistant phenotype and genotype were retained. Also in the saliva, the resistant genotype of MADTP^res^ CHIKV was maintained. Our results illustrate that the fitness of drug-resistant variants should be evaluated in both hosts to be able to select antiviral drugs with a limited risk for the spread of drug resistance by mosquitoes.

**IMPORTANCE** Because of its global reemergence and unusual morbidities associated with infection, the chikungunya virus (CHIKV) has become a substantial public health problem. However, no antivirals are currently available to treat CHIKV infections. If antiviral drugs will prove to be efficient to treat CHIKV-infected patients, it will be essential to understand if drug-resistant viruses can be transmitted from one human to another by the mosquito. We therefore orally infected *Aedes* mosquitoes with drug-resistant CHIKV variants and determined the replication and transmission levels. One of the antiviral drug-resistant CHIKV variants could efficiently replicate and disseminate in both laboratory and field-collected mosquitoes. In addition, this variant retained its drug-resistant genotype in the saliva. In contrast, the other drug-resistant variant was markedly attenuated in mosquitoes. Our results illustrate that extra caution for drug resistance should be considered when developing an antiarbovirus antiviral in order to minimize the risk of spreading drug resistance by mosquitoes.

## Abstract (precis)

Because of its global reemergence and unusual morbidities associated with infection, the chikungunya virus (CHIKV) has become a substantial public health problem. However, no antivirals are currently available to treat CHIKV infections. If antiviral drugs will prove to be efficient to treat CHIKV-infected patients, it will be essential to understand if drug-resistant viruses can be transmitted from one human to another by the mosquito. We therefore orally infected *Aedes* mosquitoes with drug-resistant CHIKV variants and determined the replication and transmission levels. One of the antiviral drug-resistant CHIKV variants could efficiently replicate and disseminate in both laboratory and field-collected mosquitoes. In addition, this variant retained its drug-resistant genotype in the saliva. In contrast, the other drug-resistant variant was markedly attenuated in mosquitoes. Our results illustrate that extra caution for drug resistance should be considered when developing an antiarbovirus antiviral in order to minimize the risk of spreading drug resistance by mosquitoes.

## INTRODUCTION

In the past decades, the world has experienced a vast increase in epidemics of arboviral infections. Due to increased global travel, trade, and urbanization, the geographic distribution of arbovirus infections has expanded and is still expanding. Arboviruses are transmitted through the bites of an infected arthropod, predominantly mosquitoes, and ticks. They are a substantial threat to the health of humans and animals worldwide, because these viruses can cause a variety of symptoms that range from mild to life-threatening diseases. One of the arboviruses causing serious health issues worldwide is chikungunya virus (CHIKV). This virus is transmitted by Aedes aegypti and Aedes albopictus mosquitoes, mostly present in tropical and subtropical regions. In the last decade, CHIKV reemerged in many parts of Africa and Asia, causing large-scale epidemics. In late 2013, the first locally transmitted infections were reported in the Americas, on the Caribbean island of Saint Martin (1). From there, the virus has further spread to neighboring countries in the Caribbean and South as well as Central America. Concomitantly, multiple imported cases in travelers returning from areas of endemicity have been reported in several European countries, Canada, and Australia. In addition, autochthonous cases have been described in Europe as well: in Italy (2007 and 2017) and in France (2010 and 2014) (2). Infections with CHIKV cause an acute disease characterized by fever, headache, and painful arthritis and usually resolve within several days. The acute stage can progress into a chronic infection in about 15 to 60% of infected patients lasting for several months or even years after the initial infection (3).

For most arboviruses, including CHIKV, there are no vaccines or antivirals available to prevent or treat infections. CHIKV-infected patients are currently given analgesics, antipyretics, and anti-inflammatory agents to alleviate their symptoms. Several molecules with *in vitro* anti-CHIKV activity have been reported (4), mostly with moderate activity and an unknown or nonspecific mechanism of action. None has progressed toward further development.

As the importance of arbovirus infections has become clearer in the last 50 years, the antiviral research field has recently embarked on the discovery of antivirals against certain arboviruses, especially dengue viruses and Zika virus. As seen with other viruses for which antiviral therapies are already available, the development of drug resistance could be a major hurdle to overcome. RNA viruses have a high mutation rate because the viral RNA-dependent RNA polymerase (RdRp) lacks proofreading activity. This results in large genetic sequence diversity. Under suboptimal antiviral pressure, resistant viral variants can rapidly become the dominant species in the virus population, as has been shown for hepatitis C virus, HIV, and influenza virus (5–7). If antiviral drugs are approved for the treatment of CHIKV-infected patients in the future, it will be very important to know how easily drug-resistant viruses can be selected in treated CHIKV patients and whether such resistant variants could be transmitted from one human to another by the mosquito vectors.

In this study, we assessed the risk of transmission of antiviral drug-resistant CHIKV by *Aedes* mosquitoes. To this end, we used two CHIKV variants resistant to antivirals with a different mechanism of action and a different barrier of resistance: T-705 (favipiravir) and MADTP (8, 9). T-705 is a broad-spectrum antiviral that is currently approved in Japan to treat influenza virus infections (10). T-705 is activated intracellularly to its ribofuranosyl 5′-triphosphate form, which is then believed to inhibit the viral RdRp. For CHIKV, the key resistance mutation is located in the nsP4 gene that encodes the RdRp, resulting in low-level resistance (8). Additional mutations in the nsP2 and nsP3 genes are required to achieve a modest level of resistance (10-fold), indicating that the resistance barrier is high for this molecule. The MADTP molecules inhibit the guanylyltransferase activity of the nsP1 protein and thereby viral RNA capping (9). In contrast to T-705, only one mutation in the nsP1 gene (P34S) is required for a fully resistant phenotype.

Both A. aegypti and A. albopictus mosquitoes were infected with wild-type (WT) or resistant CHIKV variants by infectious blood meals. We demonstrated that one of the resistant variants, MADTP^res^ CHIKV, was transmitted through the saliva expectorated by both mosquito species. Furthermore, the virus population in the mosquito saliva carried the MADTP resistance mutation in its nsP1 gene. In contrast, the dissemination and transmission of the T-705^res^ CHIKV was markedly decreased compared to WT. Our results thus clearly show the importance of studying the replication kinetics, the transmission, and the genetic stability of antiviral drug-resistant arboviruses in mosquitoes. Furthermore, this study underlines that the development of antivirals with a high barrier to resistance should be a priority for antiarbovirus therapy to minimize the risk of spreading drug-resistant viruses in the population by mosquito vectors.

## RESULTS

### Infection, dissemination, and transmission of WT and drug-resistant CHIKV.

To study the replication and transmission abilities of antiviral drug-resistant arboviruses in mosquitoes, A. aegypti Paea mosquitoes were orally infected with three CHIKV strains: (i) a wild-type CHIKV 899 strain (isolated in Mauritius in 2006, ECSA lineage) (11), (ii) a T-705^res^ CHIKV 899 strain, and (iii) a MADTP^res^ CHIKV 899 strain. The phenotype and genotype of both resistant virus strains were characterized before in detail in mammalian cell culture (8, 9). Sequence analysis of the genotype of the resistant CHIKV variants used in this study confirmed the presence of previously described mutations (see Table S1 in the supplemental material).

10.1128/mSphere.00230-18.1 TABLE S1  Mutations identified by deep sequencing in the WT and T-705^res^ and MADTP^res^ CHIKV variants. Sequences were compared to the original 899 isolate (GenBank accession no. FJ959103.1). Key resistance mutations are marked in green. Download TABLE S1, DOCX file, 0.02 MB. Copyright © 2018 Delang et al. 2018 Delang et al. This content is distributed under the terms of the Creative Commons Attribution 4.0 International license.

**FIG 1:**
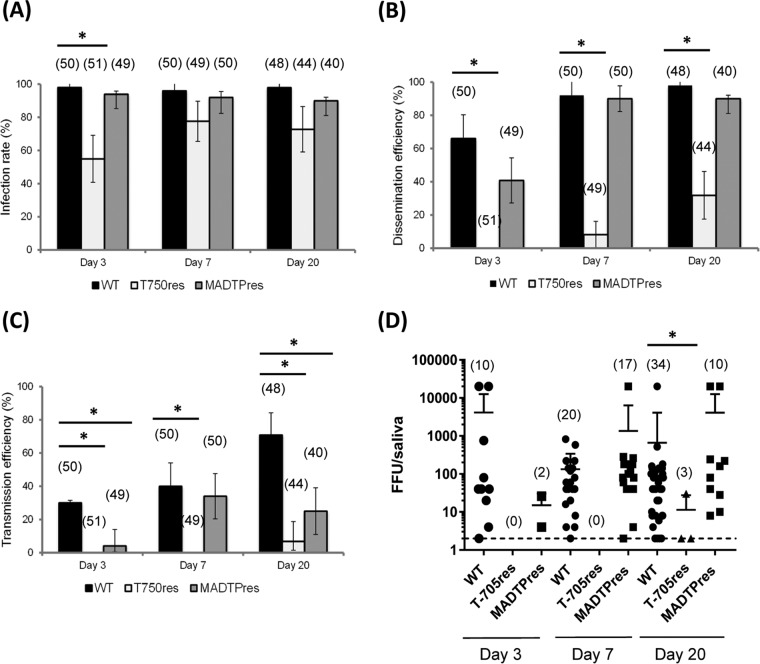
Infection, dissemination, and transmission of WT and drug-resistant CHIKV by A. aegypti Paea mosquitoes. Infection rates (A), dissemination efficiencies (B), transmission efficiencies (C), and viral titers in saliva samples (D) for A. aegypti Paea mosquitoes orally infected with WT, T-705^res^, and MADTP^res^ CHIKV. (A to C) Data (infection, disseminated infection, and transmission) are described using median values and interquartile ranges (IQR) and were analyzed using the Stata software. The number of mosquitoes tested is shown in parentheses. *, *P* < 0.05 (Fisher’s exact test). (D) Virus titers in saliva are shown as individual values; the line represents the mean value ± standard deviation (SD). The dotted line represents the detection limit. Data were analyzed with the Mann-Whitney test. The number of mosquitoes tested is shown in parentheses.

To measure the ability of mosquitoes to be infected with WT or resistant CHIKV, the infection rate (IR) was assessed at days 3, 7, and 20 after the infectious blood meal (Fig. 1A). These time points were selected as CHIKV was shown previously to be able to be transmitted in the saliva as early as 2 days postinfection (dpi) and to reach peak titers at 7 dpi (12). Twenty days p.i. was selected to study late stages of infection. After infection with CHIKV, the IR of WT and MADTP^res^ CHIKV-infected mosquitoes was >90%, starting from 3 dpi. The IR of T-705^res^ CHIKV-infected mosquitoes was significantly lower at 3 dpi (55%; Fisher’s exact test; *P* < 0.05), but reached an IR similar to that of WT CHIKV-infected mosquitoes at 7 dpi (Fisher’s exact test; *P* > 0.05). To evaluate the ability of mosquitoes to allow CHIKV to overcome the midgut barrier, the dissemination efficiency (DE) was assessed (Fig. 1B). After infection with WT CHIKV, the DE increased with the day p.i., reaching almost 100% at 7 dpi, confirming the high susceptibility of this mosquito species to CHIKV in general. The dissemination of MADTP^res^ CHIKV in mosquitoes appeared to be slower than for WT CHIKV, with a lower DE at 3 dpi compared to WT, but the DE increased to similar levels at 7 dpi. In contrast, the dissemination of T-705^res^ CHIKV in mosquitoes only started at 7 dpi (4 mosquitoes with a disseminated infection upon 49 mosquitoes tested). At 20 dpi, the dissemination of this virus was still markedly lower than both WT and MADTP^res^ CHIKV (Fisher’s exact test; *P* < 0.05).

To measure the ability of mosquitoes to allow virus to be transmitted with the saliva, the transmission efficiency (TE) was assessed (Fig. 1C). WT CHIKV was transmitted in the saliva as early as 3 dpi in 20% of the tested mosquitoes. The TE further increased along with dpi, reaching the highest level at 20 dpi (71%). The transmission of MADTP^res^ CHIKV in mosquito saliva also started at day 3 p.i., albeit at lower levels than WT CHIKV (4% versus 20%). The TE of MADTP^res^ CHIKV reached its highest level at 7 dpi (30%), which was still lower than the TE of the WT (40%) and did not increase further at a later time point. In accordance to the dissemination results, the transmission of T-705^res^ CHIKV was completely absent at 3 and 7 dpi and very low at 20 dpi compared to WT CHIKV (TE of 7% versus 71%).

The viral load determined in saliva collected from mosquitoes can estimate the intensity of viral transmission. The numbers of viral particles in saliva collected at 3 dpi were on average 3.6 log_10_ focus-forming units (FFU) and 1.2 log_10_ FFU for WT and MADTP^res^ CHIKV, respectively (Fig. 1D). For saliva collected at 7 dpi, the mean numbers of virus particles per saliva sample were 2.1 log_10_ FFU and 3.1 log_10_ FFU for WT and MADTP^res^ CHIKV. No statistically significant differences were observed between WT and MADTP^res^ CHIKV at these time points (unpaired, two-tailed *t* test with Welch’s correction). At 20 dpi, the number of viral particles in saliva was on average 1.1 log_10_ FFU for the T-705^res^ CHIKV, which was lower than the mean values of viral particles of WT CHIKV (2.8 log_10_ FFU) and MADTP^res^ CHIKV (3.6 log_10_ FFU) (Fig. 1D).

### Infection, dissemination, and transmission by field-collected mosquitoes.

**FIG 2:**
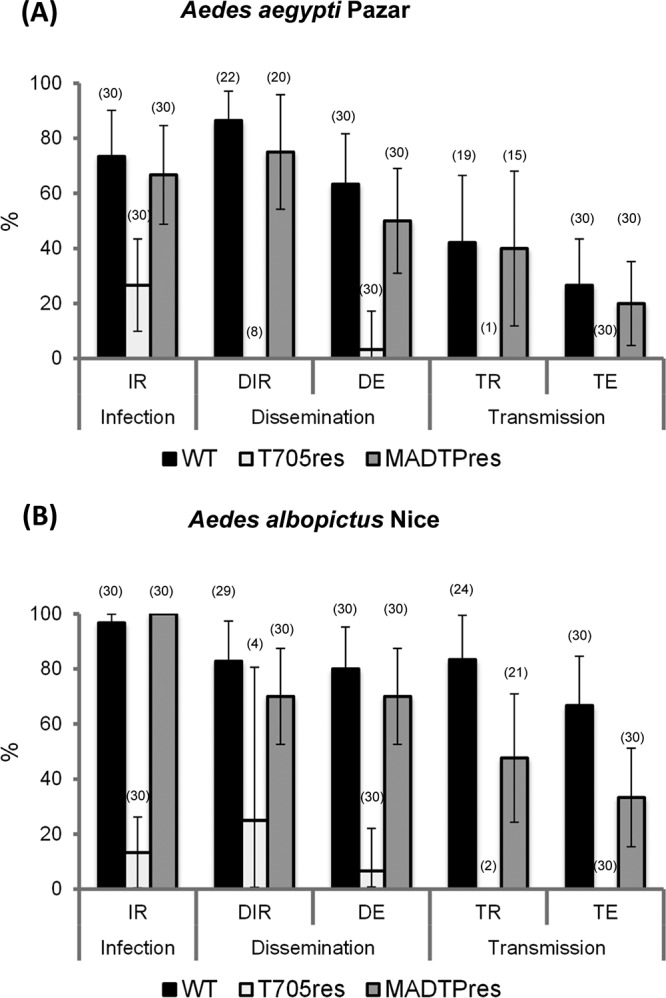
Infection, dissemination, and transmission of WT and drug-resistant CHIKV by field-collected mosquitoes. (A) Infection rates (IR), dissemination rates (DIR) and efficiencies (DE), and transmission rates (TR) and efficiencies (TE) detected at 7 dpi for A. aegypti Pazar from Turkey (F3) orally infected with the WT CHIKV 899 strain and the resistant CHIKV strains T-705^res^ and MADTP^res^. Data (infection, disseminated infection, and transmission) are described using median and interquartile range (IQR) and were analyzed using the Stata software. The number of mosquitoes tested is shown in parentheses. (B) Infection rates (IR), dissemination rates (DIR) and efficiencies (DE), and transmission rates (TR) and efficiencies (TE) detected at 7 dpi for A. albopictus from France (F10) orally infected with the WT CHIKV 899 strain and the resistant CHIKV strains T-705^res^ and MADTP^res^. Data (infection, disseminated infection, and transmission) are described using the median and interquartile range (IQR) and were analyzed using the Stata software. The number of mosquitoes tested is shown in parentheses.

The A. aegypti Paea mosquitoes used in the previous experiments have been maintained in laboratory for a long time and could thus have gone through a genetic drift from the original field-collected population. Therefore, we orally infected two field-collected *Aedes* species with WT and drug-resistant CHIKV variants: (i) A. aegypti collected in Pazar in the northeast of Turkey (AA-Pazar) and (ii) A. albopictus collected in the city of Nice in the south of France (AL-Nice). Infection rate (IR), disseminated infection rate (DIR), dissemination efficiency (DE), transmission rate (TR), and transmission efficiency (TE) were assessed at day 7 after the infectious blood meal (Fig. 2A and B), because in previous studies the peak for CHIKV transmission was observed at this time point (12). For both field-collected mosquito species, the infection and dissemination of MADTP^res^ CHIKV were similar to those of WT CHIKV, as exemplified by the IR, DIR, and DE values (Fisher’s exact test; *P* > 0.05). The transmission of MADTP^res^ CHIKV by A. albopictus AL-Nice was lower than that of the WT, whereas the rates of transmission by A. aegypti AA-Pazar were similar for both WT and MADTP^res^ CHIKV. For both AA-Pazar and AL-Nice, the IR of T-705^res^ CHIKV was lower than those of WT and MADTP^res^ CHIKV (Fisher’s exact test; *P* < 0.05). Furthermore, the dissemination of T-705^res^ CHIKV was markedly decreased in both field-collected species compared to the WT, and no transmission was observed. The data with field-collected mosquitoes thus largely confirmed the previously obtained results in the A. aegypti Paea laboratory colony.

### Evolution of the antiviral resistance of CHIKV populations in mosquitoes.

**FIG 3:**
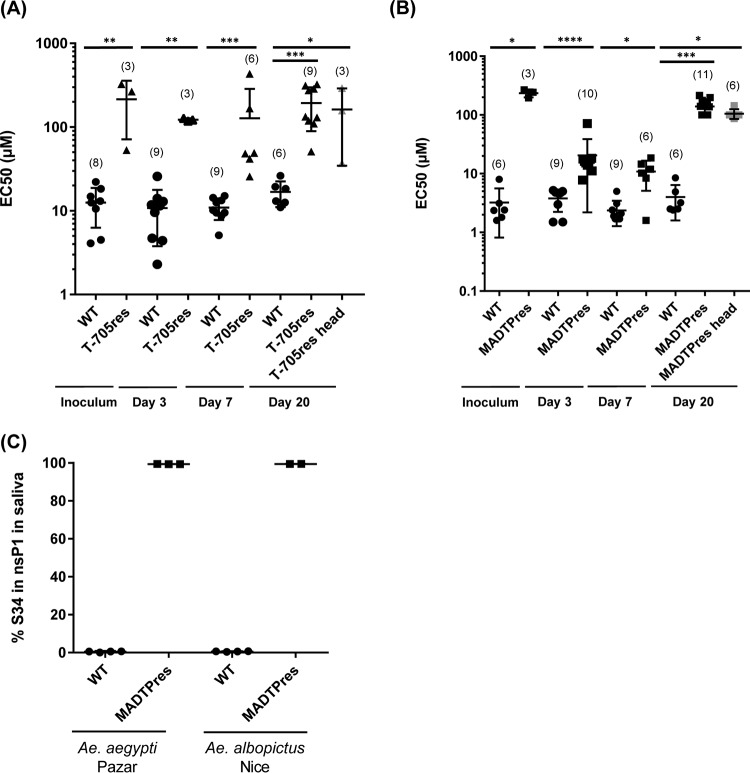
Evolution of the antiviral resistance in CHIKV populations in mosquito bodies. EC_50_s of (A) T-705 and (B) MADTP-372 for CHIKV variants in the inoculum and in individual mosquito body and head homogenates at 3, 7, and 20 dpi. The data shown are individual EC_50_s; the line represents the mean ± SD value for each condition. The number of mosquitoes tested is shown in parentheses. Data were analyzed with the Mann-Whitney test using Graphpad Prism software: *, *P* < 0.05; **, *P* < 0.001; ***, *P* < 0.0005; ****, *P* < 0.0001. (C) Percentage of serine at position 34 in the nsP1 gene in the saliva. Virus populations in the saliva collected at 7 dpi of WT- and MADTP^res^ CHIKV-infected mosquitoes were deep sequenced. The percentage of the virus population in individual saliva samples carrying a serine at position 34 in the nsP1 gene is depicted. The line represents the mean value for each group of samples.

To determine whether the virus populations in the mosquitoes were still resistant to the antiviral compounds, cytopathic effect (CPE) inhibition-based antiviral assays were performed with individual mosquito body homogenates. For all the homogenates, the same multiplicity of infection (MOI) was used (0.001) to facilitate comparison. Strikingly, the CHIKV populations in the mosquito bodies infected with T-705^res^ or MADTP^res^ CHIKV were still resistant to the antiviral effect of T-705 or MADTP-372, respectively, even following 20 days of replication in the mosquito. For the mosquitoes infected with T-705^res^ CHIKV, the 50% effective concentrations (EC_50_s) of T-705 varied between 118 and 128 µM, 26 and 431 µM, and 110 and 324 µM at 3, 7, and 20 dpi, respectively (Fig. 3A). This is approximately 12-fold higher than the EC_50_ values of the WT CHIKV populations in the mosquito bodies and similar to the fold resistance of the original T-705^res^ virus stock (8). For the mosquitoes infected with MADTP^res^ CHIKV, the EC_50_s of MADTP-372 varied between 8 and 18 µM, 2 and 19 µM, and 138 and 214 µM at 3, 7, and 20 dpi, respectively (20-, 5-, and 43-fold higher than WT) (Fig. 3B). Similar results were obtained with homogenates of mosquito heads, indicating that the resistant phenotype was not lost upon crossing the midgut barrier (Fig. 3A and B). By Sanger sequencing, the key resistance mutation P34S in the nsP1 gene could be detected in all the bodies of MADTP^res^ CHIKV-infected mosquitoes tested (*n =* 6 of each time point), whereas it was not detected in the WT CHIKV-infected mosquitoes (*n =* 3 for each time point).

To determine whether the resistant genotype would be maintained during transmission, deep sequencing analysis was performed on a selection of saliva samples. Individual saliva samples collected at 7 dpi of infected AA-Pazar and AL*-*Nice mosquitoes were passaged once on C6/36 cells. Consequently, the viral RNA was extracted and analyzed by deep sequencing. The CHIKV population in the saliva samples of MADTP^res^ CHIKV-infected mosquitoes predominantly carried the serine amino acid substitution at position 34 in nsP1 (99.4 and 99.5% of the total CHIKV populations in AA*-*Pazar and AL*-*Nice, respectively) (Fig. 3C). On the other hand, a proline was present at this position in the CHIKV population in saliva of WT CHIKV-infected mosquitoes (99.5%). These results indicate that the MADTP-resistant genotype was not lost by crossing the salivary glands barrier. Attempts to deep sequence the limited saliva samples of T-705^res^ CHIKV-infected mosquitoes were not successful, probably due to the very small amounts of virus present in these samples.

### Transmission of WT and T-705^res^ CHIKV following intrathoracic injection.

**FIG 4:**
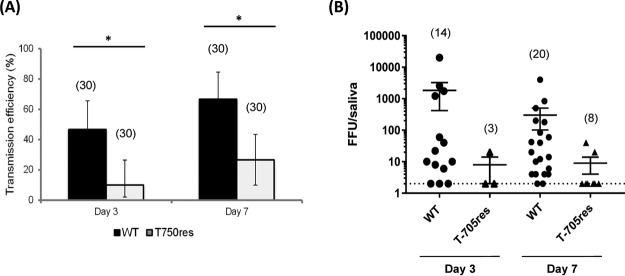
Transmission of WT and T-705^res^ CHIKV after intrathoracic injection. (A) Transmission efficiencies detected at 3 and 7 dpi for A. aegypti Paea after intrathoracic injection with 1,000 PFU of the WT CHIKV 899 strain or the resistant CHIKV strain T-705^res^. Data are described using median and interquartile range (IQR) and were analyzed using the Stata software. The number of mosquitoes tested is shown in parentheses. *, *P* < 0.05 (Fisher’s exact test). (B) Virus titers of saliva samples of individual mosquitoes. The titers are shown as individual values; the line represents the mean value ± SD. The dotted line represents the detection limit. Data were analyzed with the Mann-Whitney test. The number of mosquitoes tested is shown in parentheses.

To find out whether the midgut barrier plays a key role in the low dissemination and transmission of the T-705^res^ CHIKV strain, A. aegypti Paea mosquitoes were intrathoracically injected with 1,000 PFU of WT or T-705^res^ CHIKV, thereby circumventing the midgut barrier. Virus titers in individual saliva samples were examined at 3 and 7 dpi to assess transmission. Interestingly, the transmission efficiency of the T-705^res^ CHIKV strain proved significantly lower than the transmission efficiency of WT CHIKV (Fig. 4A). In addition, the viral titers in the saliva of T-705^res^-infected mosquitoes were lower than the titers in saliva of WT CHIKV-infected mosquitoes, although not statistically significantly lower (Fig. 4B). These data suggest that the midgut barrier is not solely responsible for the low dissemination and transmission of the T-705^res^ CHIKV variant.

### Attenuated fitness of T-705^res^ CHIKV in mosquitoes and mosquito cell culture.

**FIG 5:**
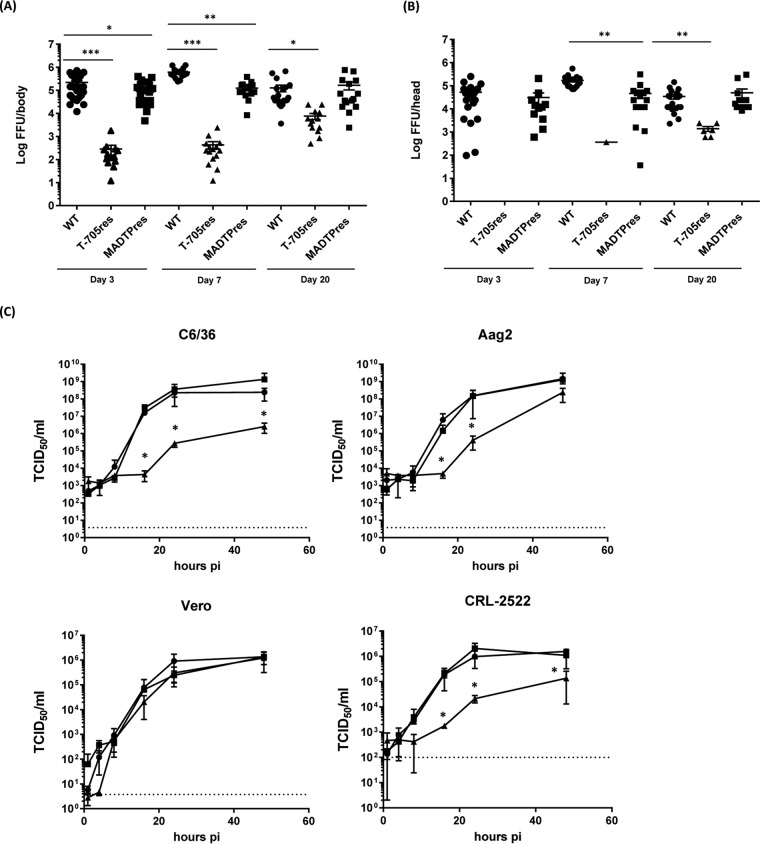
Replication fitness in mosquitoes and cell culture. (A and B) Viral titers of WT, T-705^res^ and MADTP^res^ CHIKV in (A) bodies and (B) heads of A. aegypti Paea mosquitoes detected at 3, 7, and 20 dpi. The virus titers are shown as individual values; the line represents the mean value ± SD. Data were analyzed with the Mann-Whitney test. *, *P* < 0.05; **, *P* < 0.001; ***, *P* < 0.0001. *n =* 27, 13, and 28 at 3 dpi, *n =* 17, 15, and 16 at 7 dpi, and *n =* 20, 13, and 15 at 20 dpi for bodies of WT, T-705^res^, and MADTP^res^ CHIKV-infected mosquitoes. *n =* 22, 0, and 11 at 3 dpi, *n =* 20, 1, and 17 at 7 dpi, and *n =* 20, 6, and 15 at 20 dpi for heads of WT, T-705^res^ and MADTP^res^ CHIKV-infected mosquitoes. (C) Replication kinetics of WT and drug-resistant CHIKV in mosquito and mammalian cells. *In vitro* growth curves of WT (circles), T-705^res^ (triangles), and MADTP^res^ (squares) CHIKV were determined on C6/36, Aag2, Vero, and CRL-2522 cells. The data shown are mean values ± SD from duplicates of two independent experiments. Data were analyzed by the Mann-Whitney test. The dotted line shows the detection limit. *, *P* < 0.05.

Next, we quantified the viral titers in body and head samples of individual A. aegypti Paea mosquitoes by focus-forming assays. The viral titers in both bodies and heads of the T-705^res^-infected mosquitoes were significantly lower than WT- and MADTP^res^-infected mosquito samples (Fig. 5A and B). At 3 and 7 dpi, the difference in viral titer was ~3 log_10_ FFU/head or body; at 20 dpi, the difference was smaller (~1 log_10_ FFU/head or body). These data indicate that the T-705^res^ CHIKV variant has an attenuated fitness phenotype in mosquitoes. To confirm this finding, the growth kinetics of WT, T-705^res^, and MADTP^res^ CHIKV were evaluated *in vitro* in the mammalian Vero cell line, the human skin fibroblast cell line CRL-2522, and in the mosquito cell lines C6/36 and Aag2. WT and both resistant CHIKV strains displayed very similar growth kinetics in the Vero cell line (Fig. 5C). In contrast, the growth of the T-705^res^ CHIKV strain in the mosquito cell lines proved to be markedly less efficient compared to the WT and MADTP^res^ CHIKV (Fig. 5C). Whereas the viral titers of WT and MADTP^res^ CHIKV in the supernatant were exponentially increasing between 8 and 16 hpi, the titers of the T-705^res^ CHIKV were increasing only after 16 hpi in the C6/36 and Aag2 cells. Furthermore, the viral titer at the plateau of the growth curve was significantly lower for the T-705^res^ virus than the titer of the WT and MADTP^res^ viruses (at 24 hpi, C6/36, 2.9-log_10_ difference in 50% tissue culture infective dose [TCID_50_]/ml; Aag2, 2.6-log_10_ difference in TCID_50_/ml). These data thus clearly confirm the attenuated phenotype of the T-705^res^ CHIKV variant in mosquitoes. Interestingly, the T-705^res^ CHIKV was also attenuated in the human skin fibroblast cells, which may suggest that the attenuation is not restricted to mosquitoes.

## DISCUSSION

Drug resistance is a major hurdle in the development of efficient antiviral drugs and has been studied extensively. Our knowledge on antiviral drug resistance today is largely limited to viruses that are transmitted from human to human, including hepatitis C virus, HIV, and influenza virus. For these viruses, antiviral therapy is available, and the development of resistant viral variants during antiviral therapy or the absence thereof in treated and infected patients has been well characterized (5–7). As no antivirals are currently available to treat arboviral infections, knowledge on the development and spread of antiviral drug-resistant arboviruses is lacking. In the past decades, the development and spread of drug-resistant malaria parasites by mosquitoes contributed to the failure of the 20th century Global Malaria Eradication Programme (13), illustrating that the development of drug-resistant mosquito-borne pathogens can become a serious health problem. To avoid a scenario seen with malaria and antimalaria drugs, it will thus be instrumental to understand whether mosquitoes may be able to spread antiviral drug-resistant arbovirus variants in a human population.

This study assessed the risk of transmission of antiviral drug-resistant CHIKV by its mosquito vectors. Both laboratory and field-collected *Aedes* mosquitoes were orally infected by an artificial blood meal. Our results showed that transmission of drug-resistant CHIKV by mosquitoes is possible, although it was only observed for one of the two resistant strains (the MADTP^res^) and not for the T-705^res^ variant, suggesting that different resistant viruses can have different phenotypes in mosquitoes.

For a drug-resistant arbovirus to be established in the population, transmission to another host by its vector will be required. Therefore, the resistance mutation(s) in the viral genome may not be lost before transmission to another human. In the present study, antiviral assays using the body and head samples of individual mosquitoes infected with either WT or drug-resistant CHIKV variants showed that the virus populations in the mosquitoes remained resistant to the antiviral compounds. Strikingly, the EC_50_ values of MADTP-372 at day 20 are significantly higher than the EC_50_s at days 3 and 7 (mean EC_50_ of 140 µM versus 21 and 11 µM, respectively; *P* < 0.0001 by one-way analysis of variance [ANOVA] statistical analysis). By Sanger sequencing, the presence of the key resistance mutation in nsP1 was however confirmed in all the MADTP^res^ CHIKV-infected bodies evaluated at the three time points. A possible explanation could be that the virus populations at days 3 and 7 consisted of a mixture of WT viruses and resistant viruses. Deep sequencing analysis will be needed to elucidate this.

The results of the antiviral assays clearly demonstrated that the resistance mutations were being maintained in the viral genome upon infection and dissemination in the mosquito for the studied drug-resistant chikungunya viruses. Furthermore, for the MADTP^res^ variant, we showed by deep sequencing that the resistance mutation in nsP1 was maintained in the virus population in the saliva. This could imply that when the first hurdles for antiviral resistance emergence would be taken (i.e., resistance development in a treated individual with a viral load high enough for vector infection), the spread of drug resistance in the human population may well be possible, depending on the phenotype of the resistant virus in the mosquito.

A striking observation was the markedly lower dissemination of the T-705^res^ CHIKV compared to MADTP^res^ and WT CHIKV. Intrathoracic injections of A. aegypti Paea mosquitoes showed that the transmission efficiency of the T-705^res^ CHIKV strain was still significantly lower than that of the WT CHIKV strain, despite the circumvention of the midgut barrier by the injection. The midgut barrier is thus not solely responsible for the low dissemination and transmission of T-705^res^ CHIKV. Furthermore, significantly lower titers in both bodies and heads were observed for the T-705^res^-infected mosquitoes compared to the WT-infected mosquitoes at the three time points studied. The attenuated phenotype of this variant in mosquitoes was also observed in mosquito cell culture using C6/36 and Aag2 cells. Interestingly, an attenuated phenotype was also observed in normal human skin fibroblasts. This might suggest that the attenuation is not solely restricted to mosquitoes, but is a more general feature of this resistant variant. More studies in other human cell types and in an *in vivo* model for CHIKV are needed to confirm this hypothesis.

Sequence analysis of the genotype of the T-705^res^ CHIKV used in this study confirmed the presence of previously described mutations in the nsP2, nsP3, and nsP4 genes (Table S1) (8). Of these mutations, the K291R mutation in nsP4 was shown to be the key mutation responsible for the T-705-resistant phenotype. As this lysine at position 291 in RdRp is highly conserved in positive ssRNA viruses, this mutation might be responsible for the attenuated phenotype in mosquitoes. In addition, mutations in the nsP3 hypervariable domain could be involved. For Venezuelan equine encephalitis virus (another *Alphavirus*) it was shown that phosphorylation of the hypervariable domain (HVD) of nsP3 is critical for replication in mosquito cells but not in mammalian cells (14). Interestingly, in the nsP3 gene of the T-705^res^ CHIKV, two mutations were identified in the HVD that resulted in the change of a serine into a proline and thus in a loss in phosphorylation sites. The potential contribution of these mutations to the attenuated phenotype in mosquitoes of this virus will be studied.

A limitation of this study is that the antiviral drug-resistant CHIKV variants used were selected in mammalian cell culture in the laboratory. Patient-derived virus isolates would be more clinically relevant, but as no anti-CHIKV drugs are in clinical development yet, such virus isolates do not exist at the moment. Whether drug-resistant CHIKV will emerge in an infected and treated individual is currently not known and hard to predict. This will depend on the type of antiviral drug that will receive market approval and will be affected by several characteristics of the antiviral (genetic barrier to resistance, viral fitness of resistant variants, and drug selective pressure) (15).

In conclusion, this study underlines the importance of selecting an antiviral molecule with a sufficiently high barrier of resistance for the future treatment of arbovirus-infected patients. Furthermore, our data emphasize the need to evaluate the fitness of drug-resistant arbovirus variants in both the host and the vector. These data will be necessary to select for antiviral drugs for which drug-resistant variants can ideally not be further transmitted by mosquitoes. Although the future impact of antiviral resistance development of arboviruses during treatment is difficult to predict for now, our data clearly show that extra caution for drug resistance has to be taken into account when developing an antiarbovirus antiviral in order to minimize the risk of drug resistance spread by mosquitoes.

## MATERIALS AND METHODS

### Mosquitoes.

Three *Aedes* mosquito colonies were used: (i) A. aegypti Paea (Papeete, Tahiti), (ii) A. aegypti collected in Pazar in the northeast of Turkey in February 2016 by Vincent Robert (IRD, France), and (iii) A. albopictus collected in the city of Nice in the south of France in May 2011 by Pascal Delaunay (CHU de Nice). All colonies were derived from field-collected eggs. Eggs were immersed in dechlorinated tap water for hatching. After hatching, larvae were split into pans of 150 individuals and supplied every 2 days with a yeast tablet dissolved in 1 liter of dechlorinated tap water. All immature stages were reared at 26 ± 1°C. Emerging adults were placed in different cages and were maintained at 28 ± 1°C with a light/dark cycle of 16 h/8 h at 80% relative humidity and supplied with a 10% sucrose solution. To produce eggs, females were fed three times a week on anesthetized mice (OF1 mice; Charles River Laboratories, Inc., Saint-Germain-Nuelles, France). The F3 and F10 generations were used for the infectious blood meals for A. aegypti Pazar and A. albopictus Nice, respectively. A. aegypti Paea has been maintained in the laboratory since 1994 (16).

### Cell cultures.

C6/36 (A. albopictus) cells (obtained from ATCC, CRL-1660) were maintained at 28°C in Leibovitz L-15 medium (Gibco, Illkirch Cedex, France) supplemented with nonessential amino acids (1×), 10% fetal bovine serum (FBS), 100 U/ml penicillin, and 100 µg/ml streptomycin. These cells were used for virus titration of mosquito saliva and homogenates. Vero E6 (green monkey kidney) cells (obtained from ATCC; CRL-1586) were used for production of parental CHIKV stocks and stock titrations and were maintained at 37°C in 5% CO_2_ in Dulbecco’s modified Eagle’s medium (DMEM) (Gibco) with 10% FBS, 100 U/ml penicillin, and 100 µg/ml streptomycin.

### Viruses.

CHIKV Indian Ocean strain 899 (GenBank accession no. FJ959103.1) was generously provided by C. Drosten (University of Bonn, Germany) (11). The MADTP-resistant CHIKV and T-705-resistant CHIKV strains were previously selected in cell culture using a clonal resistance selection method (8, 9) and were kindly provided by J. Neyts (University of Leuven, Belgium). The virus stocks were produced on Vero cells. Virus production of the resistant CHIKV stocks was performed in the presence of 90 µM MADTP-314 or 100 µM T-705 for the MADTP-resistant and T-705-resistant viruses, respectively. Serial dilutions were used to determine the titer of viral stocks by plaque assay as described before (17).

### Compounds.

MADTP-314 and MADTP-372 were a kind gift of M. J. Pérez-Pérez (University of Madrid, Spain); T-705 (favipiravir) was a kind gift of J. Neyts (University of Leuven, Belgium). Compounds were dissolved in analytical-grade dimethyl sulfoxide (DMSO).

### Experimental infections of mosquitoes.

Infection assays were performed with 7- to 10-day-old females starved 24 h prior to infection in a biosafety level 3 (BSL-3) laboratory.

### (i) Infectious blood meals.

Mosquitoes were allowed to feed for 15 min through a piece of pork intestine covering the base of a Hemotek feeder containing the infectious blood meal maintained at 37°C. The blood meal was composed of 1/3 viral supernatant to 2/3 washed rabbit erythrocytes isolated from arterial blood and ATP at a final concentration of 10^−3^ M. The titer of the infectious blood meals was 10^6.8^ PFU/ml for all CHIKV variants. Engorged females were separated and incubated under controlled conditions (28 ± 1°C, relative humidity of 80%, light/dark cycle of 16 h/8 h). At 3, 7, and 20 dpi, vector competence was assessed based on three phenotypes: (i) viral infection of the midgut, (ii) viral dissemination from the midgut into mosquito general cavity, and (iii) transmission potential with virus detected in mosquito saliva. Saliva was collected as described before (12). Briefly, wings and legs were removed from each individual, and its proboscis was inserted into a 20-µl tip containing 5 µl FBS. After 45 min, saliva-containing FBS was expelled in 45 µl of Leibovitz L-15 medium (Gibco) for titration. Experimentally induced salivation is widely used to demonstrate the transmission of pathogens ingested by hematophagous insects (18). Following salivation, the head of each mosquito was removed, and both the body and the head were homogenized individually in 300 µl of 2% Leibovitz L-15 medium. Homogenates were stored at −80°C before processing. Transmission efficiency (TE) was calculated as the overall proportion of females that had infectious saliva (i.e., among all tested females with or without a disseminated infection). TE was then broken down into two intermediate indices. Dissemination efficiency (DE) was calculated as the proportion of females with infected head tissues (i.e., in which the virus successfully disseminated from the midgut). Transmission rate (TR) was defined as the proportion of females with infectious saliva among those that developed a disseminated infection. Therefore, TE equals the product of DE and TR (19).

### (ii) Intrathoracic inoculations of mosquitoes.

One-week-old females were inoculated with WT CHIKV and T-705-resistant CHIKV at equal titers using the protocol described by Rosen and Gubler (20). Each mosquito received 166 nl of viral suspension corresponding to 10^4^ PFU of each virus by using the Nanoject II Auto-Nanoliter injector (Drummond Scientific). Inoculated mosquitoes were incubated under controlled conditions (28 ± 1°C, relative humidity of 80%, light/dark cycle of 16 h/8 h). At 3 and 7 dpi, vector competence was largely assessed as described above. Following the collection of saliva, the entire body of the mosquito was homogenized individually in 300 µl of 2% Leibovitz L-15 medium, after which the homogenates were stored at −80°C before processing.

### Virus titration and quantification.

The presence of infectious virus particles in mosquito bodies, heads, and saliva extracts were determined by focus-forming assay in C6/36 cells as previously described (21). Briefly, 96-well plates were seeded with cells, and each well was inoculated with 50 µl of saliva extract or head/body homogenate and incubated for 1 h at 28°C. Then, cells were overlaid with a 1:1 mix of carboxymethyl cellulose and Leibovitz L-15 medium supplemented with 10% FBS and 1.5× an antibiotic-antifungal solution (Gibco Life Technologies, Inc., France). After 3 days of incubation, cells were fixed for 20 min at room temperature with 3.7% formaldehyde, washed three times in phosphate-buffered saline (PBS), and incubated for 15 min with 0.5% Triton X-100 in PBS. Cells were then incubated for 1 h with a hyperimmune ascitic fluid specific to CHIKV as the primary antibody, washed three times with PBS, and incubated for 1 h at room temperature with a goat anti-mouse conjugate as the second antibody (Bio-Rad, Hercules, CA). The number of focus-forming units was determined under a fluorescence microscope. The data were analyzed qualitatively (i.e., presence or absence of infectious virus in heads/bodies) and quantitatively for saliva samples and some body and head samples.

### Deep sequencing of the viral RNA.

From each saliva sample, amplified for one passage on C6/36 cells, CHIKV was precipitated using 10% of polyethylene glycol 8000 (Sigma). RNA was then isolated with TRI reagent and chloroform and processed with the NEBNext Ultra II RNA Library Prep kit (Illumina). Multiplex oligonucleotides (Illumina) were used during the library process. Sequencing of the libraries diluted to 1 nM was performed on a NextSeq 500 sequencer (Illumina) with a NextSeq 500 Mid Output kit v2 (Illumina) (151 cycles). Adaptors and low-quality bases were trimmed from reads using cutadapt (https://cutadapt.readthedocs.io/en/stable/), and reads with low quality were eliminated. Reads were mapped on CHIKV 899 strain genome using Bowtie2 (http://bowtie-bio.sourceforge.net/bowtie2/index.shtml) to generate sam files, allowing one mismatch between reads and their target. sam files were processed to produce bam indexed files using the samtools package. Graphs were generated from these bam files using Integrative Genomic Viewer software. (http://software.broadinstitute.org/software/igv/).

### Antiviral assays.

Vero cells were seeded in 96-well tissue culture plates at a density of 2.5 × 10^4^ cells/well in 100 µl 2% Vero medium and were allowed to adhere overnight. Next, a compound dilution series of T-705 and MADTP-372 was prepared, after which the cultures were infected with an inoculum at an MOI of 0.001 (prepared individually for each mosquito homogenate) in 100 µl 2% medium (total volume). On 5 dpi, the plates were processed using the MTS-PMS [3-(4,5-dimethylthiazol-2-yl)-5-(3-carboxymethoxyphenyl)-2-(4-sulfophenyl)-2H-tetrazolium–phenazine methosulfate] method as described by the manufacturer (Promega, Leiden, Netherlands). The 50% effective concentration (EC_50_) was determined using logarithmic interpolation.

### Replication kinetics.

To measure viral replication fitness, growth curves were studied in A. albopictus C6/36 cells, A. aegypti Aag2 cells, human skin fibroblast cells, and mammalian Vero cells. Confluent cell monolayers were prepared in 96-well plates and inoculated with viruses simultaneously in duplicates at an MOI of 1 PFU/cell. Cells were incubated for 1 h in appropriate conditions. The viral inoculum was removed, and cell monolayers were washed 3 times with PBS to eliminate unbound virus. Two hundred microliters of medium supplemented with 2% FBS was added, and cells were incubated at 28 or 37°C depending on the cell line. At various times (0, 4, 8, 16, 24, and 48 h) postinoculation, supernatants were collected and titrated by endpoint dilution on Vero cells. The TCID_50_ values were calculated using the method of Reed and Muench (22).

### Statistical analysis.

Rates (infection, disseminated infection, and transmission) were described using the median and interquartile range (IQR). Statistical analyses were conducted using the Stata software (StataCorp LP, Texas, and United States). *P* values of <0.05 were considered significant. Virus titers and EC_50_s were described using mean values and standard deviations. Statistical analyses (two-tailed, unpaired *t* test) were conducted using the GraphPad Prism software. *P* values of <0.05 were considered significant.

### Ethics statement.

The Institut Pasteur animal facility has received accreditation from the French Ministry of Agriculture to perform experiments on live animals in compliance with the French and European regulations on care and protection of laboratory animals. This study was approved by the Institutional Animal Care and Use Committee (IACUC) at the Institut Pasteur.
