# Supplementary material for: Differential Transmission of Antiviral Drug-Resistant Chikungunya Viruses by Aedes Mosquitoes
Source: mSphere. 2018 Aug 22;3(4):e00230-18. doi: 10.1128/mSphere.00230-18 (PMC6106055; doi:10.1128/mSphere.00230-18)
Supplement: TABLE S1 [file sph004182631st1.docx]

**Supplemental information**

**Supplemental Table 1. Mutations identified by deep sequencing in the WT, T-705^res^ and MADTP^res^ CHIKV.** Sequences were compared to the original 899 isolate (Genbank [FJ959103.1](https://www.ncbi.nlm.nih.gov/nuccore/FJ959103.1)). The key resistance mutations are marked in green.

| **WT CHIKV** | | | | |
| --- | --- | --- | --- | --- |
| **position** | **CHIKV899** | **WT** | **AA change** | **gene** |
| 330 | G | A | R85H | nsP1 |
| 822 | C | T | P249L | nsP1 |
| 1247 | C | T | L391F | nsP1 |
| 3309 | A | G | Y543C | nsP2 |
| 10867 | G | A | D292N | E1 |
| **T-705^res^ CHIKV** | | | | |
| **position** | **CHIKV899** | **T-705^res^** | **AA change** | **gene** |
| 330 | G | A | R85H | nsP1 |
| 822 | C | T | P249L | nsP1 |
| 1247 | C | T | L391F | nsP1 |
| 1872 | A | G | K49R | nsP2 |
| 1920 | A | G | E80G | nsP2 |
| 2273 | A | G | N198D | nsP2 |
| 2487 | T | C | V269A | nsP2 |
| 2894 | T | C | S405P | nsP2 |
| 3005 | A | G | N442D | nsP2 |
| 3012 | A | G | K444R | nsP2 |
| 3309 | A | G | Y543C | nsP2 |
| 3546 | A | G | E622G | nsP2 |
| 3936 | A | G | K752R | nsP2 |
| 4167 | A | G | D31G | nsP3 |
| 4794 | A | G | E240G | nsP3 |
| 5109 | T | C | F345S | nsP3 |
| 5121 | T | C | V349A | nsP3 |
| 5189 | G | T | D372Y | nsP3 |
| 5334 | A | G | N420D | nsP3 |
| 5423 | T | C | S450P | nsP3 |
| 5486 | T | C | S471P | nsP3 |
| 5647 | A | G | Opal524W | nsP3 |
| 6425 | G | A | A254T | nsP4 |
| 6537 | A | G | K291R | nsP4 |
| 6641 | A | G | I326V | nsP4 |
| 6695 | A | G | T344A | nsP4 |
| 6875 | A | G | I404V | nsP4 |
| **MADTP^res^ CHIKV** | | | | |
| **position** | **CHIKV899** | **MADTP^res^** | **AA change** | **gene** |
| 176 | C | T | P34S | nsP1 |
| 330 | G | A | R85H | nsP1 |
| 822 | C | T | P249L | nsP1 |
| 1247 | C | T | L391F | nsP1 |
| 3309 | A | G | Y543C | nsP2 |
| 10867 | G | A | D292N | E1 |
